# PGLN: A newly identified amino phosphoglycolipid species in *Thermus thermophilus* HB8

**DOI:** 10.1016/j.bbrep.2022.101377

**Published:** 2022-11-02

**Authors:** Naoki Nemoto, Masahiko Kawaguchi, Kei Yura, Haruo Shimada, Yoshitaka Bessho

**Affiliations:** a Faculty of Advanced Engineering, Chiba Institute of Technology, 2-17-1 Tsudanuma, Narashino, Chiba, 275-0016, Japan; b Graduate School of Humanities and Sciences, Ochanomizu University, 2-1-1 Otsuka, Bunkyo, Tokyo, 112-8610, Japan; c Center for Interdisciplinary AI and Data Science, Ochanomizu University, 2-1-1 Otsuka, Bunkyo, Tokyo, 112-8610, Japan; d Graduate School of Advanced Science and Engineering, Waseda University, 3-4-1 Okubo, Shinjuku, Tokyo, 169-8555, Japan; e BioChromato, Inc, 1-12-19 Honcho, Fujisawa, Kanagawa, 251-0053, Japan; f School of Life Sciences, Tokyo University of Pharmacy and Life Sciences, 1432-1 Horinouchi, Hachioji, Tokyo, 192-0392, Japan; g Graduate School of Agricultural and Life Sciences, The University of Tokyo, 1-1-1 Yayoi, Bunkyo, Tokyo, 113-8657, Japan; h RIKEN SPring-8 Center, Harima Institute, 1-1-1 Kouto, Sayo, Hyogo, 679-5148, Japan

**Keywords:** Amino phospholipid, Glucosamine, Glycolipid, Polarlipid, Thermophiles, Phospholipid synthetic pathway

## Abstract

*Thermus thermophilus* has several minor lipid molecules with structures that have not been described yet. In this study, we identified a new lipid molecule in *T. thermophilus* HB8 with an amino group at the polar head, by detecting lipid spots with HPTLC and mass spectrometry. The structure of the lipid resembles an amino sugar phospholipid, except for the glucosamine that lacks an acetyl group. We named this amino phosphoglycolipid PGLN, and proposed its synthetic pathway from a precursor, phosphatidyl-glyceric alkylamine. The primary amine structure of PGLN may contribute to high temperature adaptation through electrostatic interactions between the head groups.

## Highlights

• No amino phospholipid has been identified in *T. thermophilus* HB8 so far.
• PGLN is discovered by detecting lipid spots with HPTLC and mass spectrometry.
• PGLN is a newly identified amino phosphoglycolipid without an acetyl group.
• PGLN may play an important role in high temperature adaptation.

## 1 Introduction

*Thermus thermophilus* is a Gram-negative aerobic bacterium originally obtained in 1968 at Mine Onsen hot spring, Izu Peninsula, Japan [1]. This bacterium can grow in a high temperature environment around 80 °C and its proteins are thermostable; hence, these proteins have been utilized for molecular function studies and structure determinations [[2], [3], [4]]. The lipid molecules in the cell membrane of *T. thermophilus* have numerous characteristics suitable for a high temperature environment compared with the standard phospholipids of mesophilic bacteria, such as the addition of iso or ante-iso branched fatty acids, the difference in the number of hydrophobic fatty acid side chains (three instead of two), a glycolipid as the major lipid and so on [5]. In addition, a monosaccharide is attached to a *T. thermophilus* phospholipid and forms a phosphoglycolipid. The structures of the three main lipid molecules, namely two glycolipid molecules (GL1 and GL2) and one phosphoglycolipid molecule (PGL), have been determined [5,6].

**Fig. 1:**
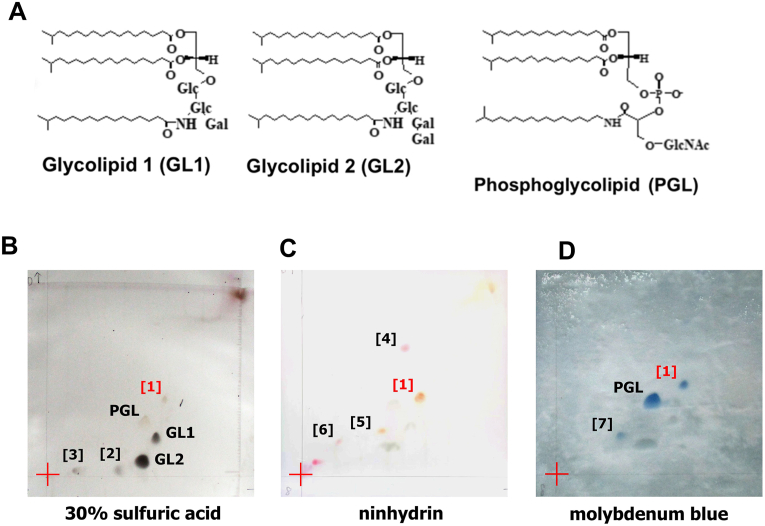
Detection of primary amine and phosphoric acid groups attached to polar lipid groups by HPTLC. (A) Structures of the three major lipids (GL1, GL2, and PGL) in *T. thermophilus* HB8. (B) Spots detected by 30% sulfuric acid under white light. (C) Spot-1 appeared upon ninhydrin treatment. (D) Two evident spots (Spot-1 and PGL) appeared with the molybdenum blue spray reagent. Blue spots signify the detected phospholipids. (For interpretation of the references to color in this figure legend, the reader is referred to the Web version of this article.)

GL1 (Fig. 1A left) has a glycero-backbone with two ester-linked acyl-groups, and three hexoses: glucose, glucosamine, and galactose. The amino group of the glucosamine binds covalently to another acyl-group. GL2 (Fig. 1A center) has one more galactose attached to the galactose of GL1, resulting in a glycolipid molecule with four sugar groups. PGL (Fig. 1A right) has a glycero-backbone with two ester-linked acyl-groups and one phosphate group. The hydroxyl group at position 2 of another glycero-backbone forms a covalent bond with the phosphate group. N-acetylglucosamine and an alkylamine bind covalently to the glycero-backbone, thus forming the PGL phosphoglycolipid molecule. Numerous minor lipid molecules in *T. thermophilus* have been detected by TLC [7], but not been identified yet.

The *T. thermophilus* enzymes involved in the biosynthesis of branched fatty acids and ester-bond formation have been characterized or predicted based on amino acid sequence similarity [[8], [9], [10], [11], [12]]. The glycolipid groups of GL1 and GL2 are presumed to be synthesized by transferring a monosaccharide to diacylglycerol, as reported in other organisms [13]. However, the enzymes that catalyze the reaction to form the third hydrophobic chain specific for *T. thermophilus* and the reaction to replace the polar head of the phosphoglycolipid (PGL) have not been identified.

In *T. thermophilus*, polar lipids have only been identified in PGL, and lipids with amino groups such as phosphatidylserine (PS) or phosphatidylethanolamine (PE) have not been reported. Lipid molecules with electric charges are likely to contribute to the functions of membrane proteins. The phospholipid molecules attract charged proteins to the membrane, establishing their functions at the surface of the membrane. Especially, amino phospholipid molecules are reportedly involved in protein function expression and are considered to be indispensable lipid molecules [[14], [15], [16]]. For instance, PE, with an amino group at its polar head, interacts with amino acid residues in autophagy-related proteins at the surface of the membrane [14]. The polar head groups of PE also reportedly interact with specific amino acid residues in the yeast mitochondrial *bc*1 complex of the respiratory chain [15]. PS, with an amino group at its polar head, is known to play important roles in blood coagulation reactions [16].

Despite the importance of lipid molecules with amino groups at the polar heads, PS and PE have not been identified in *T. thermophilus*. Therefore, we speculated that a thorough investigation of ligands in the *T. thermophilus* HB8 (type strain) cell should reveal novel lipid molecules with an amino group at their polar heads. In this study, we addressed this question using mass spectrometry and identified a new lipid molecule with an amino group at its polar head.

## 2 Materials and methods

### 2.1 Culture conditions and lipid extraction

*T. thermophilus* HB8 was grown at 80 °C for 7 days without shaking in *Thermus* nutrient medium [17]. The culture was centrifuged at 4000 g for 10 min using a swing rotor, and the cells were washed twice with PBS(−). Lipids were extracted from the cells by a modified Bligh and Dyer method (ThermoLipo Inc.) [18]. The extracted lipids were dissolved at 10 μg/μL in chloroform-methanol (2:1, v/v) and used for the following analyses.

### 2.2 Separation and detection of lipids

Thin-layer chromatography (TLC) was performed on Silica Gel 60 High Performance TLC (HPTLC) plates (Merck KGaA, Germany), which were activated by an incubation at 120 °C for 30 min before use. The plates were spotted with 10 μL of whole-cell lipid extract. For two-dimensional TLC (2-D TLC), chloroform-methanol-water (65:25:4, v/v) was used for the vertical direction (first dimension) and chloroform-acetic acid-methanol-water (80:15:12:4, v/v) was used for the horizontal direction (second dimension). Total lipid spots on HPTLC plates were detected by spraying the plates with 30% (w/v) H_2_SO_4_ and charring at 150 °C for 30 min, or with a *p*-anisaldehyde solution and charring at 120 °C for 10 min [19]. The spots were visualized under natural light. For phospholipid detection, spots were stained with molybdenum blue spray reagent (Sigma-Aldrich, Germany) [20]. For glycolipid detection, spots were stained with an anthrone solution and incubated at 110 °C for 10 min [21]. For amino lipid detection, spots were stained with a ninhydrin solution and incubated at 100 °C for 5 min [22]. All reagents, besides the molybdenum blue spray reagent, used for these lipid-spot staining reactions were purchased from FUJIFILM Wako, Japan.

### 2.3 Mass spectrometry

The target lipid was recovered from TLC plates after one-dimensional development with chloroform-acetic acid-methanol-water (80:15:12:4, v/v), without treatment with any labeling reagents. The spot with a retention factor (Rf) value of approximately 0.42 on the silica gel plate was removed and extracted by the modified Bligh and Dyer method. The sample was dried and dissolved in 200 μL of ethanol-THF (4:1, v/v). The total lipid fraction was dissolved in the same solvent. The target lipid and total lipid fractions were analyzed by a quadrupole time-of-flight mass spectrometer (qTOFMS, COMPACT; Bruker, MA, USA) equipped with an ESI ion source, using a direct infusion technique. The flow rate was maintained at 180 μL/h. Nitrogen was used as the nebulizing and desolvation gas. The temperature and the flow rate of the desolvation gas were 200 °C and 3 L/min, respectively. Mass spectra (MS) were acquired in a negative ion mode in the mass range of *m/z* 50–2500.

## 3 Results

### 3.1 Detection of lipid species by HPTLC

Lipid detection with 30% sulfuric acid resulted in three spots, named Spot-1, Spot-2, and Spot-3, in addition to the three major lipid molecules, PGL, GL1, and GL2 (Fig. 1B). The TLC spots of the three major lipid molecules were assigned by comparing their positions with those in the previous reports [5,23].

Among the six spots detected in 30% sulfuric acid, the ninhydrin treatment for the detection of a lipid molecule with an amino group only colored Spot-1 (orange) (Fig. 1C). A substrate with one ninhydrin molecule is reportedly colored orange, and one with two ninhydrin molecules is purple [24]. Therefore, these color differences assist in the molecular structure estimation (see discussion). Due to the high sensitivity of the ninhydrin coloring reaction, three more spots were observed. We numbered these spots as 4 to 6 from top to bottom in Fig. 1C. Spot-4 and Spot-6 were colored purple and Spot-5 was orange. These spots were not detected under 30% sulfuric acid conditions (Fig. 1B), suggesting that the amounts of these lipids were low.

Spot-1, in addition to PGL, was colored by molybdenum blue spray, indicating that both lipids have a phosphate group (Fig. 1D). Spot-1 was also colored by anthrone and *p*-anisaldehyde, similarly to PGL (Fig. S1). These results suggest that Spot-1 is a lipid molecule with similar characteristics to PGL, containing amino and phosphate groups (Table 1).

**Table 1:** Comparison between newly detected Spot-1 and PGL.

Reagent	Detected lipid species	Spot-1	PGL
30% sulfuric acid	total lipid	**+**	**+**
ninhydrin	amino lipid	**+**	**−**
molybdenum blue spray	phospholipid	**+**	**+**
anthrone	glycolipid	**+**	**+**
*p*-anisaldehyde	total lipid	**+**	**+**

### 3.2 Mass spectrometry of Spot-1

**Fig. 2:**
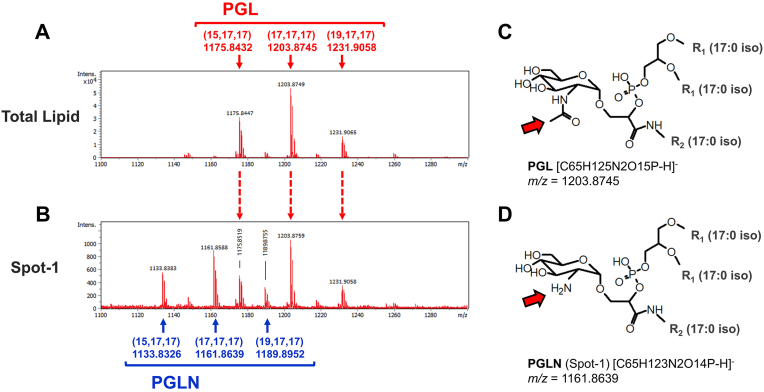
Mass spectra and expected molecular structures. Mass spectra of (A) the total lipid and (B) Spot-1. Theoretical [M − H]^-^ values are above/below the spectra. (C) A molecular structure of PGL that corresponds to the spectrum in (A). (D) A molecular structure of PGLN (Spot-1) that is expected from the spectrum in (B). The difference between PGL and PGLN is indicated by red arrows in (C) and (D). In these figures, R_1_ (17:0 iso) stands for CO(CH_2_)_13_CH(CH_3_)_2_ and R_2_ stands for (17:0 iso): (CH_2_)_14_CH(CH_3_)_2_. (15, 17, 17) is the shortened description of (R_1_,15:0 iso; R_1_, 17:0 iso; R_2_, 17:0 iso), (17, 17, 17) is of (R_1_, 17:0 iso; R_1_, 17:0 iso; R_2_, 17:0 iso), and (19, 17, 17) is of (R_1_, 19:0 iso; R_1_, 17:0 iso; R_2_, 17:0 iso). The length of the carbon chain in the parentheses is commutative. (For interpretation of the references to color in this figure legend, the reader is referred to the Web version of this article.)

Based on the fact that Spot-1 was colored by molybdenum blue spray, we assumed the existence of a phosphate moiety in the structure, and analyzed it with a qTOFMS system in the negative ion mode. The total lipid fraction of *T. thermophilus* HB8 was measured as a control. The *m/z* peak at 1175.8447 corresponded to the theoretical peak for the molecular-related ion ([M − H]^-^) of PGL with one branched fatty acid 15:0 iso chain and two branched fatty acid 17:0 iso chains (15, 17, 17; 1175.8432) (Fig. 2A). The *m/z* peak at 1203.8749 corresponded to the theoretical peak for the [M − H]^-^ of PGL with three branched fatty acid 17:0 iso chains (17, 17, 17; 1203.8745) (Fig. 2A and C). The *m/z* peak at 1231.9065 corresponded to the theoretical peak for the [M − H]^-^ of PGL with one branched fatty acid 19:0 iso chain and two branched fatty acid 17:0 iso chains (19, 17, 17; 1231.9058) (Fig. 2A). The evident peaks of Spot-1 in Fig. 2B; namely, *m/z* 1133.8383, 1161.8588, and 1189.8755, did not stand out among the peaks of the total lipid molecules (Fig. 2A). Three peaks of PGL (1175.8519, 1203.8759, and 1231.9058) were also detected in Fig. 2B, but they were likely the result of contamination derived from the extraction process.

## 4 Discussion

The HPTLC results indicated that Spot-1 is an amino phospholipid molecule with phosphate and amino groups on the polar head portion. The Rf value of two-dimensional HPTLC and the color suggested that the structure of the Spot-1 molecule is similar to that of PGL. The amino group in Spot-1 is likely to be attached to a monosaccharide of the polar lipid head, because the orange color elicited by ninhydrin is reportedly derived from an amino sugar [[24], [25], [26]]. The results of the HPTLC and mass spectrometry analyses suggested that Spot-1 is a lipid molecule with a phosphate group and a glucosamine attached to the branched fatty acid hydrocarbon chains. The theoretical [M − H]^-^ values (1133.8326, 1161.8639, 1189.8952) based on the assumed molecular structure match those of Spot-1 (Fig. 2B and D). Hence, we named this amino phosphoglycolipid molecule PGLN, because Spot-1 resembles the PGL molecule, except for a glucosamine that lacks an acetyl group. The polar lipids of other *Thermus* species reportedly contain an amino phospholipid named PLN, which was detected near PGL by two-dimensional TLC [27]. We assume that PGLN identified in this report corresponds to PLN. In *Thermus filiformis*, a lipid molecule was identified by electrospray ionization mass spectrometry (ESI-MS) and NMR analysis [28], and has the same headgroup as PGLN identified here in *T. thermophilus* HB8. Therefore, PGLN likely exists widely among the bacteria of the *Thermus* genus.

**Fig. 3:**
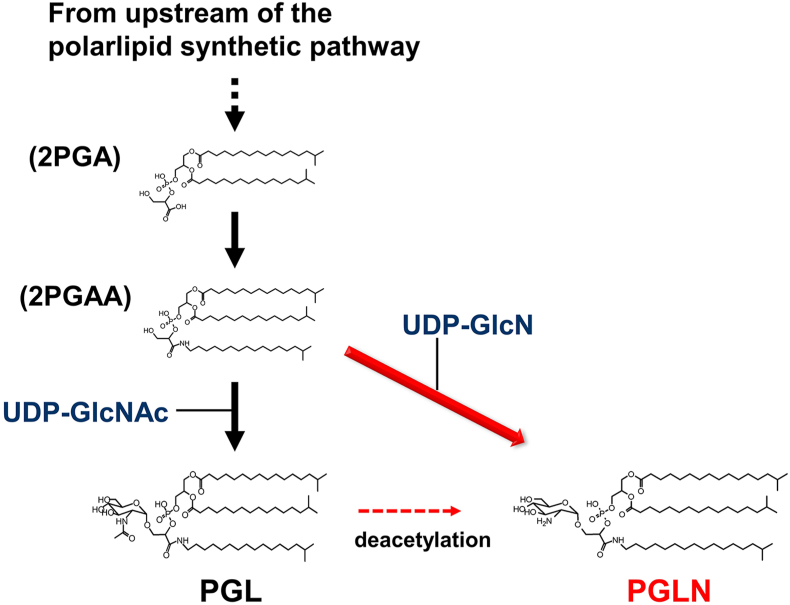
Possible biosynthetic pathways of PGL and PGLN (Spot-1) molecules in *Thermus thermophilus* HB8. The details of the pathway without PGLN were drawn in the page version PWBA0004 of the ThermusQ website [12]. The black arrows are the paths drawn in ThermusQ. The red arrow is the predicted path based on the current study. (For interpretation of the references to color in this figure legend, the reader is referred to the Web version of this article.)

*T. thermophilus* HB8 synthesizes a branched-chain fatty acid with a branched amino acid at the end of the hydrocarbon chain, which is further processed to a phospholipid in the glycerophospholipid synthetic pathway (Fig. 3). The bacteria of the *Thermus* genus probably have a pathway to attach another branched chain fatty amine to the phosphate group of the phospholipid, which would produce phosphatidyl-glyceric alkylamine (2PGAA). The attachment of UDP-N-acetylglucosamine (UDP-GlcNAc) is likely to form PGL, while that of UDP-glucosamine (UDP-GlcN), instead of UDP-GlcNAc, to 2PGAA is the probable path to the formation of PGLN. The difference between PGL and PGLN is the acetylation of the amino group of the attached glucosamine (Fig. 2C and D). The deacetylation of PGL may form PGLN, but PGLN has been reported in other *Thermus* species, suggesting that the product was not an experimental artefact or the result of degradation by deacetylation.

A study of the effects of growth temperature on the lipid composition in *Thermus* sp. strains revealed that the amounts of major phospholipids including PGL decrease and those of amino phospholipids increase under high temperature culture conditions [29]. The relatively abundant concentration of PGLN, one of the amino phospholipids, in the current study may be ascribed to the high temperature (80 °C) of the culture conditions. Electrostatic interactions between a positively charged amino group in one of the lipid molecules that is covalently bonded to glucosamine and a phosphate group in another lipid molecule with a negatively charged polar head may bridge the two molecules and, in effect, decrease the membrane fluidity, thus contributing to the resistance against high temperature.

In this study, an amino phosphoglycolipid was newly identified from *T. thermophilus* HB8, which is a model organism for thermophiles. This finding not only elucidates the biosynthetic pathway of phospholipids, but also clarifies the physiological roles of biological membrane molecules at high temperatures.

## Author contributions

NN and YB conceptualized this paper; NN, MK, and HS performed the experiments; NN, MK, HS, and YB analyzed the experimental data. NN, KY, HS, and YB wrote the manuscript. All authors have agreed to publish this version of manuscript.

## Declaration of competing interest

KY, HS, and YB are shareholders of ThermoLipo Inc.

## Data Availability

No data was used for the research described in the article.
