# Supplementary material for: PGLN: A newly identified amino phosphoglycolipid species in Thermus thermophilus HB8
Source: Biochem Biophys Rep. 2022 Nov 2;32:101377. doi: 10.1016/j.bbrep.2022.101377 (PMC9636437; doi:10.1016/j.bbrep.2022.101377)
Supplement: Multimedia component 1 [file mmc1.pdf]

## Supplementary Figure S1

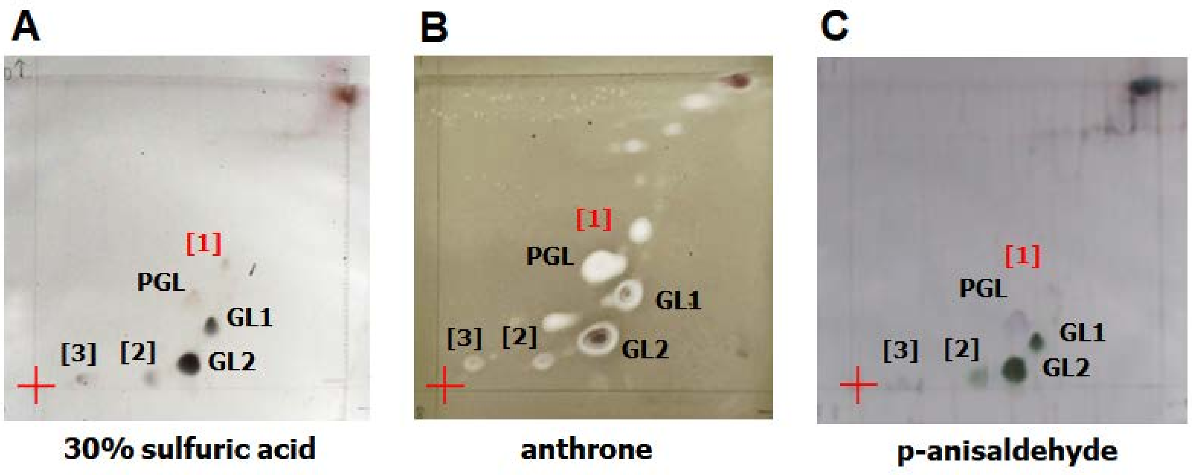


**Figure S1.** Detection of primary amine and phosphoric acid groups attached to the polar lipid group by HPTLC. (A) Spots detected by 30% sulfuric acid under white light. This figure is the same as Figure 1B. (B) Spot detection by anthrone reagent. GL1 and GL2 are dyed brown and PGL and Spot-1 are white. (C) Spot detection by *p*-anisaldehyde reagent. GL1 and GL2 are dyed green and PGL and Spot-1 are purple.
